# Adjunctive Photoactivated Chromophore for Keratitis-Corneal Cross-Linking for Refractory Pseudomonas aeruginosa Keratitis During Pregnancy: A Case Report

**DOI:** 10.7759/cureus.114207

**Published:** 2026-08-09

**Authors:** Ioanna Gardeli, Efthymios Karmiris, Tryfon Rotsos, Vasileios Panagoulis, Panagiotis Stavrakas

**Affiliations:** 1 Department of Ophthalmology - Cornea and Transplant, General Hospital of Athens "Georgios Gennimatas", Athens, GRC; 2 Department of Ophthalmology, National and Kapodistrian University of Athens, Athens, GRC; 3 Department of Ophthalmology, Democritus University of Thrace, Alexandroupoli, GRC; 4 Department of Ophthalmology, General Hospital of Athens "Georgios Gennimatas", Athens, GRC

**Keywords:** corneal melting, infection in pregnancy, pack-cxl, pseudomonas keratitis, refractory corneal ulcer

## Abstract

Management of severe infectious keratitis during pregnancy is challenging because rapid control of a sight-threatening infection must be balanced against potential fetal exposure to antimicrobial agents and surgical interventions. Photoactivated chromophore for keratitis-corneal cross-linking (PACK-CXL) has been proposed as an adjunctive treatment for infectious keratitis associated with progressive stromal melting.

A 34-year-old woman at 19 weeks of gestation presented with a two-day history of painful visual deterioration in her left eye. She was a contact lens wearer. Slit-lamp examination revealed a 5.0 × 3.5-mm paracentral corneal ulcer with a wet, necrotic appearance, an associated 3.5 × 3.5-mm stromal infiltrate, and corneal melting. Marked anterior chamber inflammation and a 0.5-mm hypopyon were present. Best-corrected visual acuity was 20/20 in the right eye and 20/200 in the left eye. Cultures of corneal scrapings and the contact lenses yielded *Pseudomonas aeruginosa*, whereas polymerase chain reaction testing for fungi and *Acanthamoeba* was negative. Following consultation with the obstetric team, intensive topical amikacin and vancomycin, topical atropine, and intravenous ceftazidime were administered. Although the hypopyon resolved and anterior chamber inflammation substantially decreased, stromal infiltration and melting persisted after 14 days of treatment. Adjunctive PACK-CXL was therefore performed using 0.1% riboflavin and ultraviolet-A irradiation at 3.0 mW/cm² for 30 minutes. The ulcer and infiltrate subsequently regressed, with complete resolution of the corneal melting. At three months, the ulcer had healed completely, leaving a paracentral stromal scar that did not significantly involve the visual axis. Best-corrected visual acuity improved to 20/28. Therapeutic penetrating keratoplasty was avoided, and the pregnancy progressed to an uncomplicated delivery.

Adjunctive PACK-CXL was associated with arrest of corneal melting and satisfactory anatomical and visual recovery in a pregnant patient with refractory *P. aeruginosa* keratitis. However, the reproductive safety of riboflavin-ultraviolet-A cross-linking has not been established. Its use during pregnancy should therefore be considered only in exceptional, sight-threatening circumstances following multidisciplinary assessment and informed evaluation of the potential maternal benefits and unknown fetal risks.

## Introduction

Infectious keratitis is an ophthalmic emergency that may progress rapidly to stromal destruction, corneal perforation, endophthalmitis, and permanent visual loss. Contact lens wear is an important risk factor for bacterial keratitis, particularly infection caused by Pseudomonas aeruginosa. This organism may induce rapid tissue destruction through direct microbial invasion, secretion of proteolytic enzymes, and stimulation of an intense host inflammatory response [1-3].

The management of infectious keratitis becomes particularly complex during pregnancy because both the maternal visual prognosis and potential fetal exposure to pharmacological treatment must be considered. Although topical ophthalmic medications generally result in substantially lower systemic exposure than oral or intravenous administration, absorption through conjunctival vessels and the nasolacrimal mucosa is not negligible. Treatment should therefore be individualized and undertaken in consultation with the treating obstetric team. Eyelid closure and nasolacrimal occlusion following topical administration may further reduce systemic absorption [4].

Severe infectious keratitis during pregnancy therefore presents a significant clinical dilemma. Sight-preserving treatment must be initiated promptly, while the potential fetal risks associated with medications, ultraviolet-A exposure, anesthesia, and surgical intervention must also be considered. Management requires an individualized, multidisciplinary assessment that balances the immediate maternal benefits against the uncertain fetal risks.

Photoactivated chromophore for keratitis-corneal cross-linking (PACK-CXL) combines a photosensitizing chromophore, most commonly riboflavin, with ultraviolet-A irradiation. The photochemical reaction may exert an antimicrobial effect while simultaneously increasing stromal resistance to enzymatic degradation [5-7]. PACK-CXL may therefore be particularly useful in infectious keratitis complicated by progressive corneal melting.

Nevertheless, the available clinical evidence remains heterogeneous and of low certainty. PACK-CXL should consequently be regarded as an adjunctive or rescue treatment rather than a routine replacement for appropriate antimicrobial therapy [8,9].

It is important to distinguish elective corneal collagen cross-linking for corneal ectatic disorders from PACK-CXL performed as an exceptional rescue intervention for sight-threatening infectious keratitis. In the former setting, cross-linking is generally avoided during pregnancy because reproductive safety data are limited [10]. In the latter setting, the potential maternal benefit of preserving corneal integrity may outweigh the theoretical fetal risk.

We report the use of adjunctive PACK-CXL in a woman at 19 weeks of gestation who developed severe contact lens-associated P. aeruginosa keratitis with persistent stromal infiltration and melting despite intensive, culture-directed antimicrobial treatment. The novelty and educational value of this case lie in the use of PACK-CXL during pregnancy as an exceptional sight-saving rescue intervention aimed at preserving corneal integrity and avoiding or deferring emergency therapeutic penetrating keratoplasty.

## Case presentation

A 34-year-old woman at 19 weeks of gestation presented to the emergency department with a two-day history of ocular pain and progressive visual deterioration in her left eye. She was a contact lens wearer. Her medical history was significant for psoriatic arthritis, which was in remission and did not require treatment at the time of presentation. She was not receiving corticosteroids, conventional immunosuppressive agents, or biological therapy; therefore, treatment-related immunosuppression was not considered a contributing factor.

**Figure 1:**
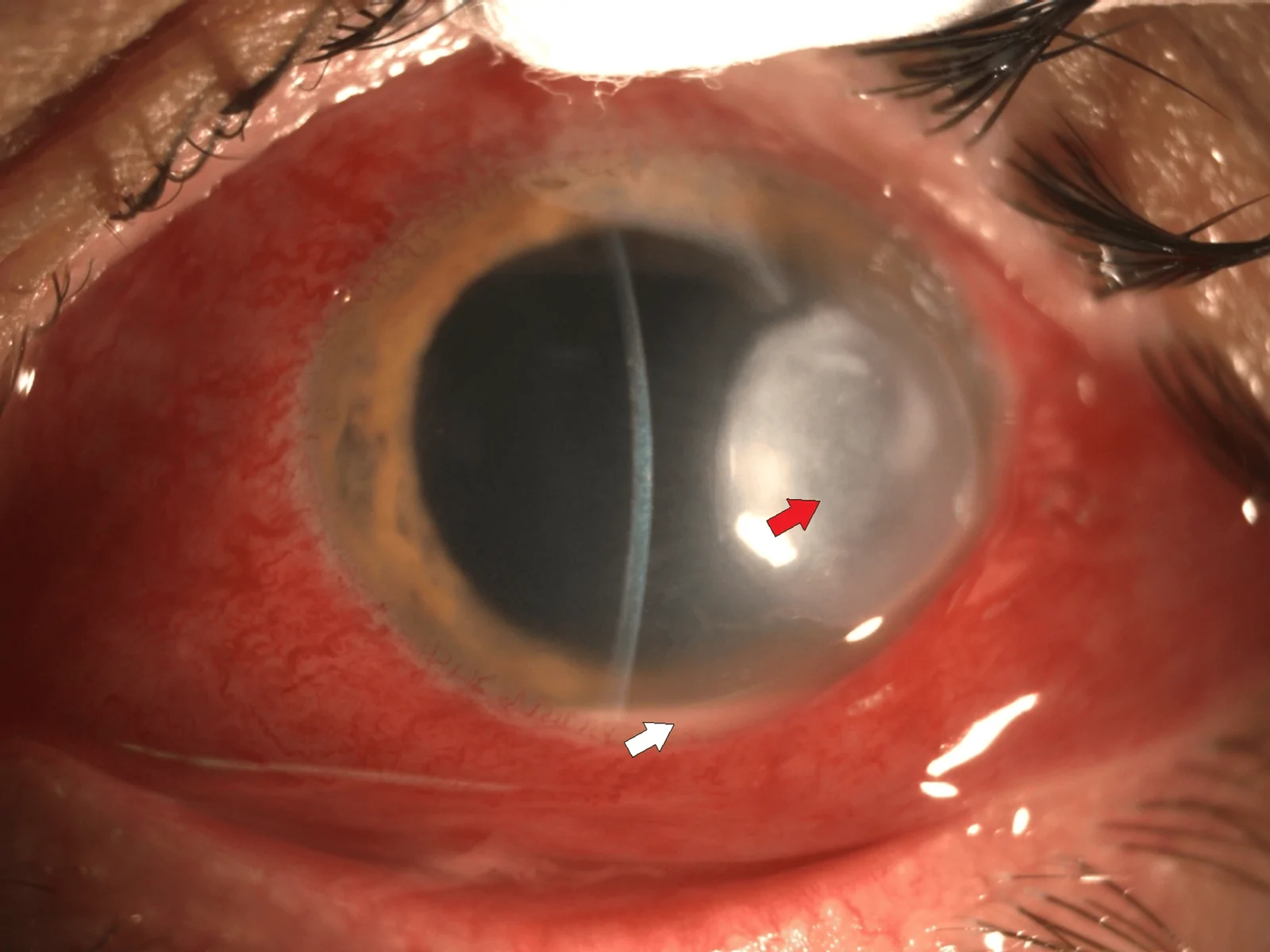
Slit lamp examination at presentation Paracentral ulcer 5.0 × 3.5 mm, with a wet appearance and clear margins (red arrow) and an associated 3.5 × 3.5 mm dense stromal infiltrate with corneal melting (yellow arrow) as well as 0.5 mm hypopyon (white arrow).

**Figure 2:**
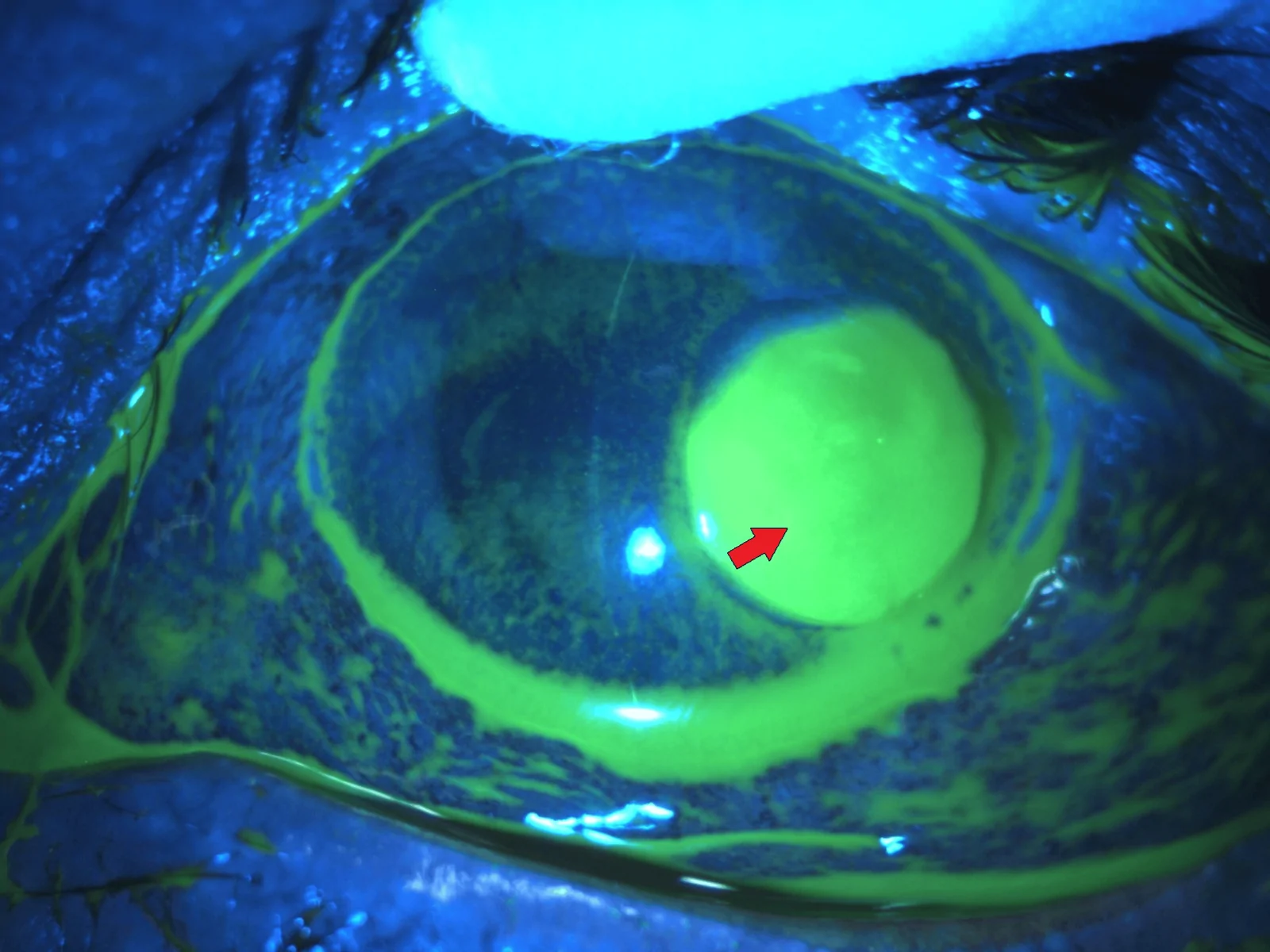
Slit lamp examination at presentation Paracentral ulcer 5.0 × 3.5 mm, fluorescein staining (red arrow).

Best-corrected visual acuity was 20/20 in the right eye and 20/200 in the left eye. Slit-lamp examination of the left eye revealed a paracentral epithelial defect and a corneal ulcer measuring 5.0 × 3.5 mm, with a wet, necrotic appearance. An associated deep stromal infiltrate measuring 3.5 × 3.5 mm and corneal melting were present. The percentage of corneal thinning was not objectively quantified. The anterior chamber demonstrated marked inflammatory activity and a 0.5-mm hypopyon. Additional findings included upper and lower eyelid edema, conjunctival congestion, and chemosis (Figures 1, 2). 

Corneal scrapings were obtained from the peripheral margins of the lesion for microbiological culture and antimicrobial susceptibility testing. Polymerase chain reaction analysis of the corneal samples was performed for fungi and Acanthamoeba. The patient’s contact lenses were also submitted for culture.

Following consultation with the attending obstetricians, intensive empirical antimicrobial treatment was initiated. The regimen consisted of topical amikacin every 15 minutes for the first three hours and hourly around the clock thereafter, topical vancomycin hourly during the day and every two hours overnight, and topical atropine. Intravenous ceftazidime was administered at a dose of 2 g three times daily for three days, followed by 1 g three times daily.

Given the pregnancy and the availability of alternative antimicrobial agents, topical fluoroquinolones and systemic doxycycline were not selected. In the event that emergency penetrating keratoplasty became necessary, systemic mannitol and acetazolamide were also planned to be avoided unless their administration became essential for the preservation of maternal ocular integrity.

Cultures of both the corneal scrapings and contact lenses yielded Pseudomonas aeruginosa. Antimicrobial susceptibility testing demonstrated susceptibility to amikacin and tobramycin and susceptibility to ceftazidime at increased antimicrobial exposure. Polymerase chain reaction testing was negative for fungi and Acanthamoeba. On the basis of these findings, the initial topical and systemic antimicrobial regimen was continued.

**Figure 3:**
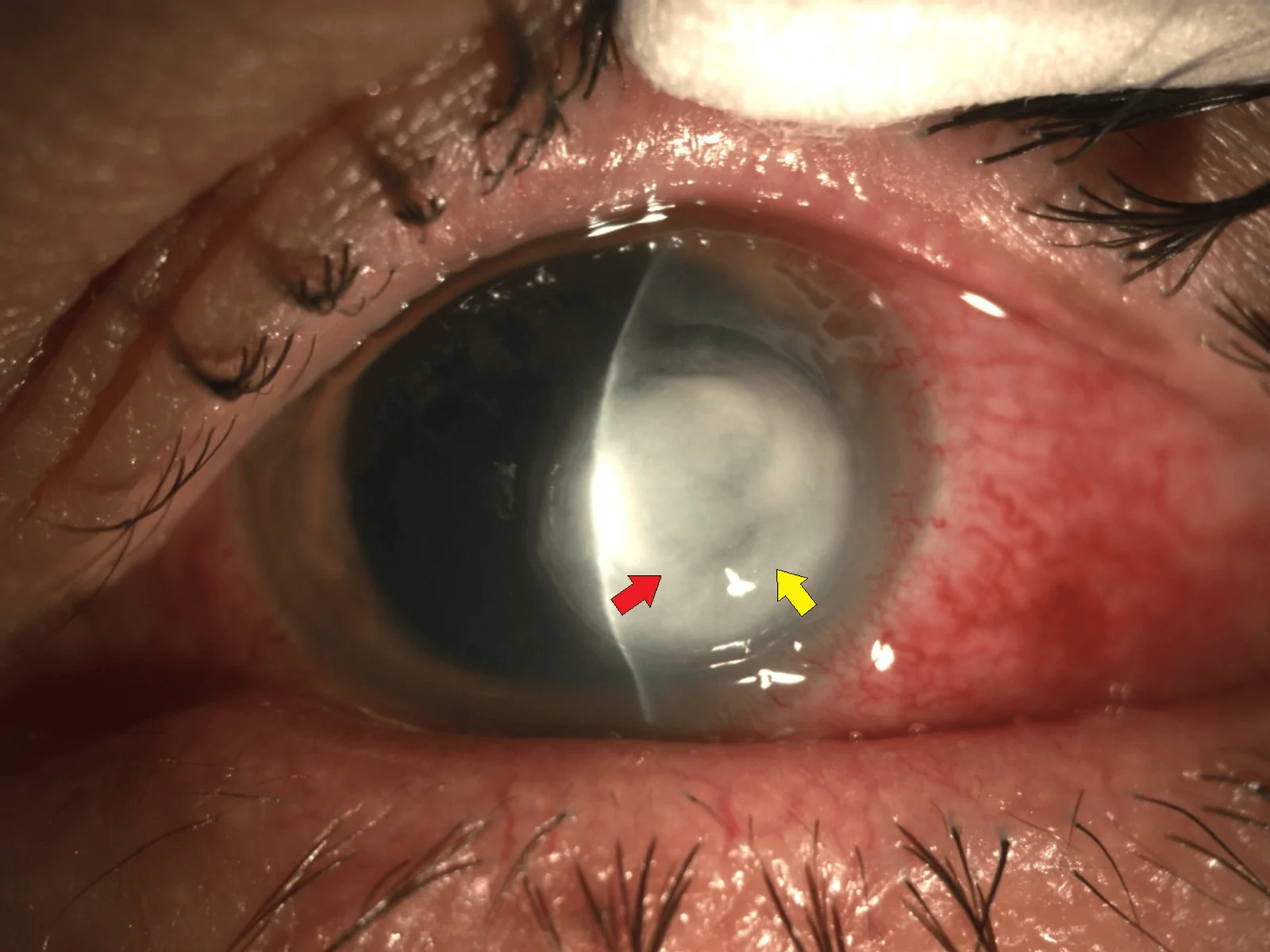
Clinical presentation after 14 days of treatment Despite the resolution of the hypopyon, the corneal ulcer only partially resolved, measuring 3.7 x 3.5 mm (red arrow), while the stromal infiltrate and corneal melting persisted (yellow arrow).

**Figure 4:**
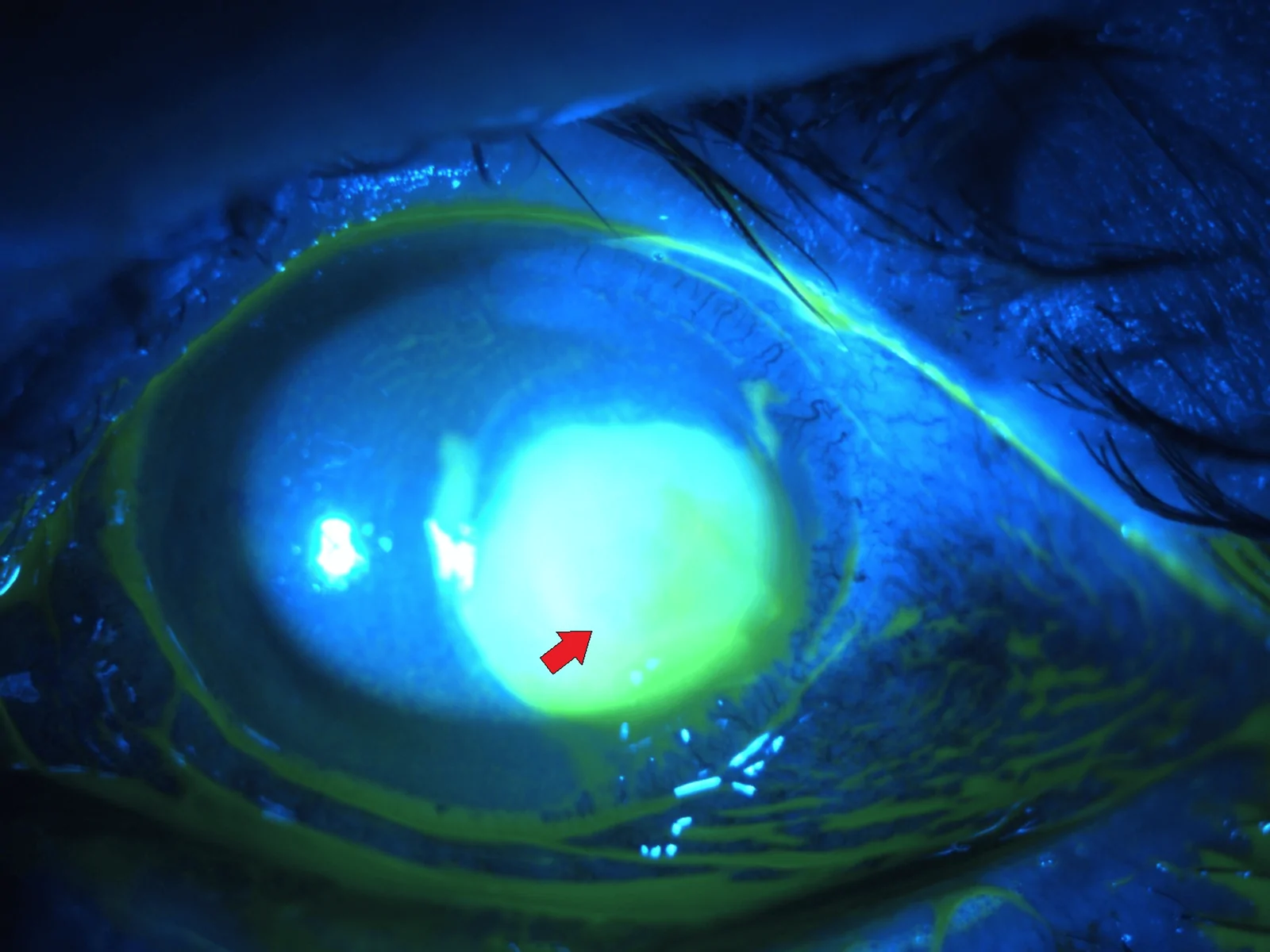
Clinical presentation after 14 days of treatment Residual corneal ulcer, fluorescein staining (red arrow).

After 14 days of treatment, the hypopyon had resolved, and anterior chamber inflammation had decreased substantially. Nevertheless, the corneal ulcer had only partially regressed, measuring 3.7 × 3.5 mm, while the stromal infiltrate and corneal melting persisted (Figures 3, 4). 

Because of the continuing risk of corneal perforation, irreversible visual loss, and loss of ocular integrity, adjunctive PACK-CXL was considered in an attempt to stabilize the corneal stroma. The resolution of the hypopyon and substantial reduction in anterior chamber inflammation suggested partial control of the active infection; however, persistent stromal infiltration and melting indicated continuing structural deterioration despite 14 days of intensive, culture-directed antimicrobial therapy. Therapeutic penetrating keratoplasty was discussed as the principal surgical rescue option should progression or perforation occur. After being informed of its potential benefits and risks, the patient declined this option and signed a written informed refusal. Other potential rescue options included tissue adhesive application in the event of a small perforation, amniotic membrane transplantation, and conjunctival flap surgery. PACK-CXL was therefore selected as a less invasive rescue intervention intended to preserve corneal integrity and avoid or defer emergency therapeutic penetrating keratoplasty. The potential maternal benefits and uncertain fetal risks of this exceptional, off-label procedure were evaluated following multidisciplinary consultation with the obstetric team and discussed in detail with the patient. Written informed consent specifically for PACK-CXL treatment was obtained before the procedure.

Residual epithelium overlying and immediately surrounding the lesion was debrided. A 0.1% riboflavin ophthalmic solution was subsequently applied to the cornea, followed by ultraviolet-A irradiation at 3.0 mW/cm² for 30 minutes, according to the conventional Dresden irradiation protocol.

**Figure 5:**
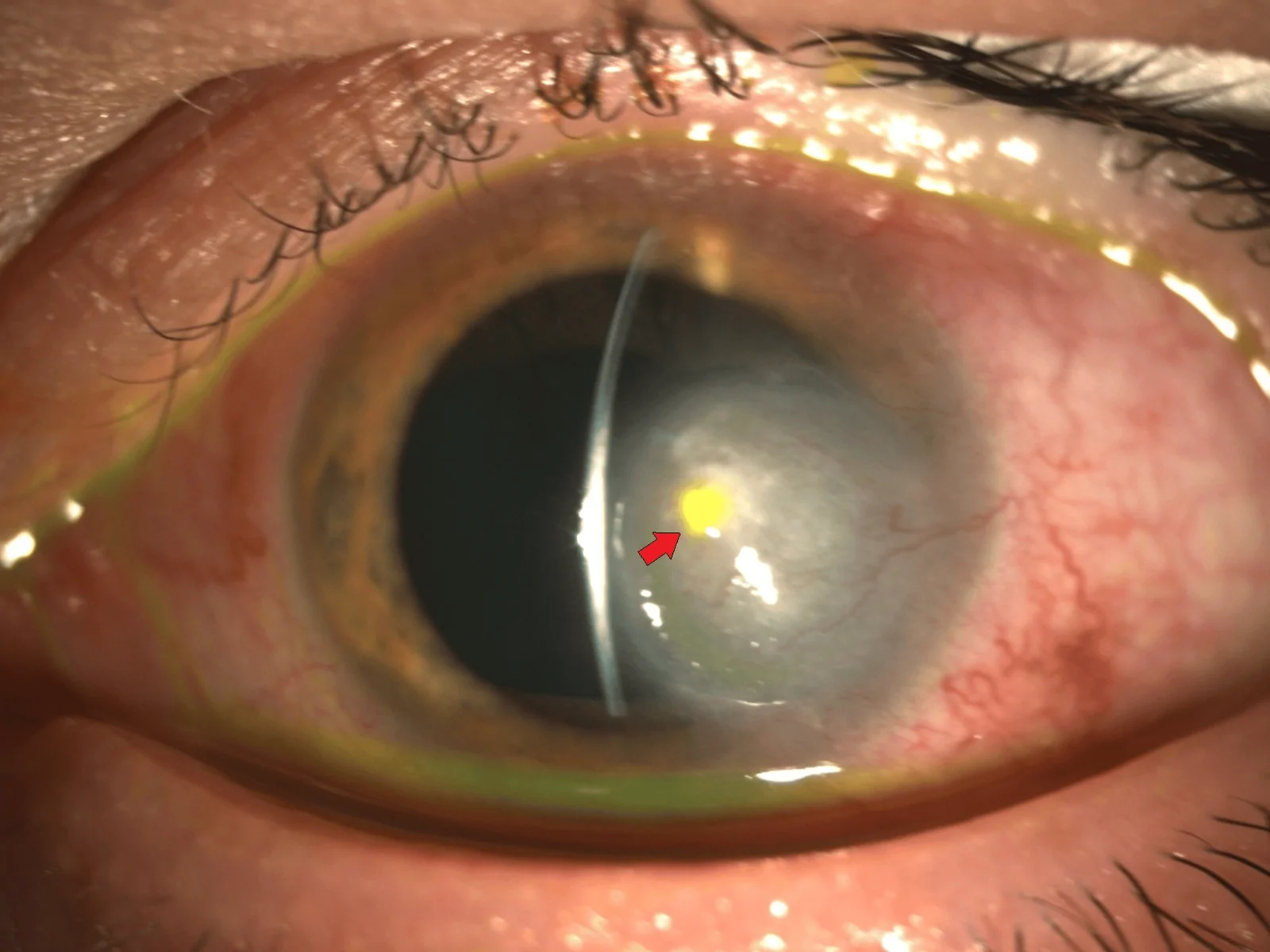
Clinical presentation two months after treatment with PACK-CXL Gradual regression of the ulcer (red arrow). PACK-CXL: photoactivated chromophore for keratitis-corneal cross-linking.

**Figure 6:**
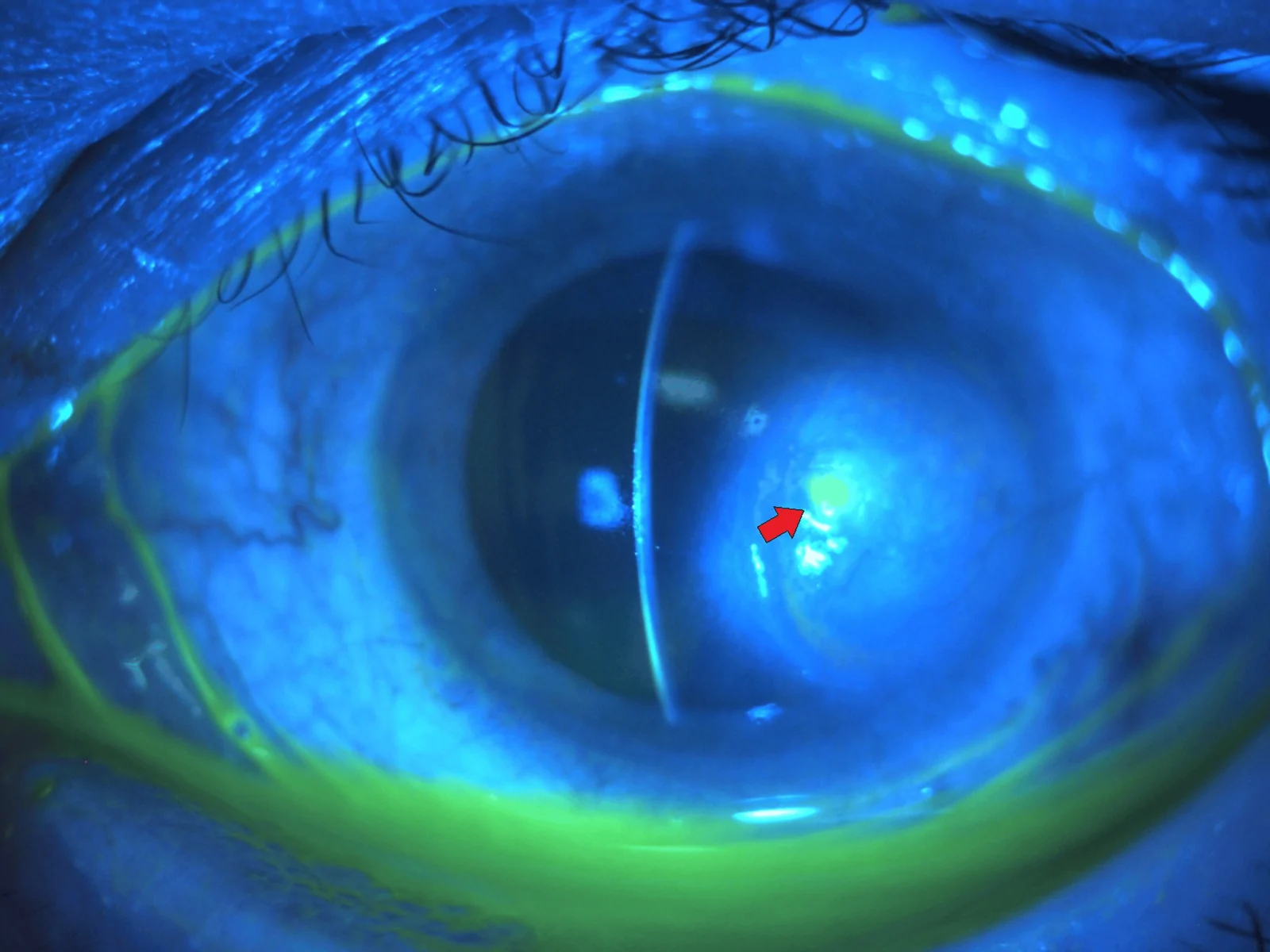
Clinical presentation two months after treatment with PACK-CXL Gradual regression of the ulcer, 0.2 x 0.2 mm, fluorescein staining (red arrow). PACK-CXL: photoactivated chromophore for keratitis-corneal cross-linking.

The patient was discharged the following day with topical tobramycin four times daily. During the subsequent weeks, the epithelial defect and stromal infiltrate gradually decreased, with complete arrest and regression of the corneal melting (Figures 5, 6). 

**Figure 7:**
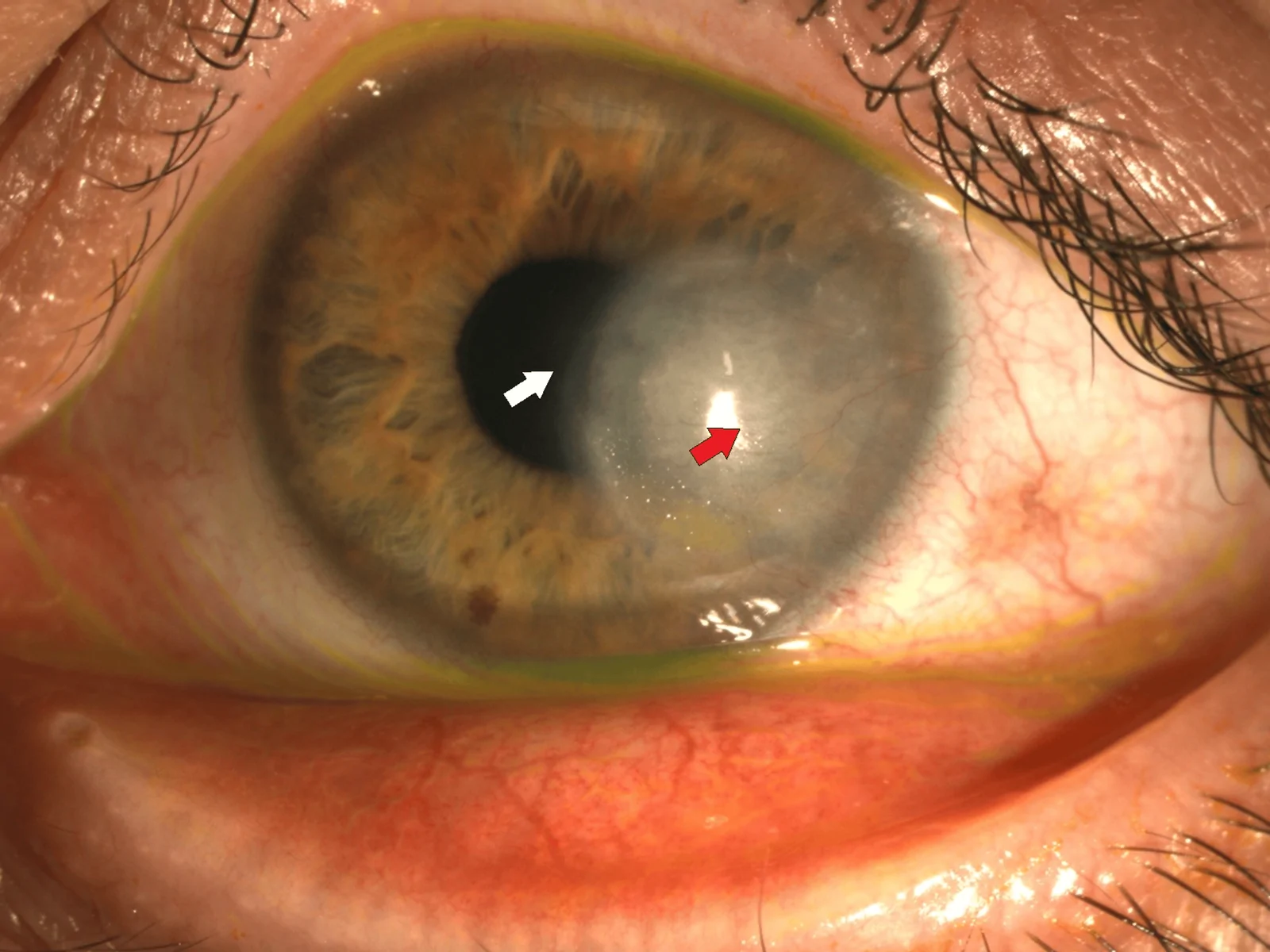
Clinical presentation four months after treatment with PACK-CXL Complete resolution of the corneal ulcer with residual paracentral scar (red arrow). Minimal involvement of the visual axis (white arrow). PACK-CXL: photoactivated chromophore for keratitis-corneal cross-linking.

**Figure 8:**
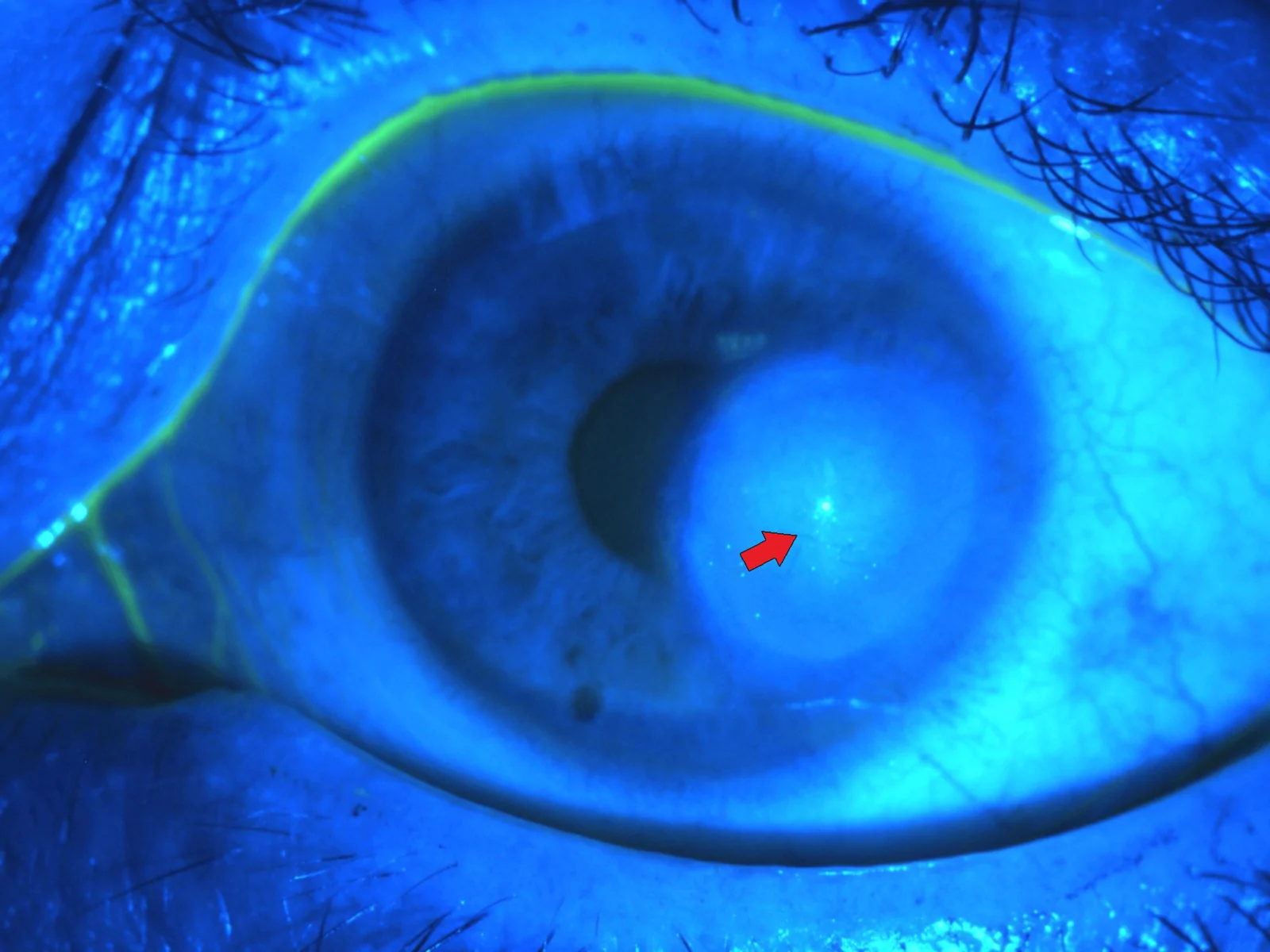
Clinical presentation four months after treatment with PACK-CXL Complete resolution of the corneal ulcer with residual paracentral scar. No fluorescein staining observed (red arrow). PACK-CXL: photoactivated chromophore for keratitis-corneal cross-linking.

Three months after PACK-CXL, the corneal ulcer had healed completely. A residual paracentral stromal scar remained but did not significantly involve the visual axis (Figures 7, 8).

Best-corrected visual acuity improved to 20/28 in the affected eye. Therapeutic penetrating keratoplasty was therefore avoided. The pregnancy continued without reported complications and culminated in an uncomplicated delivery.

## Discussion

This case illustrates the considerable therapeutic challenges associated with severe bacterial keratitis during pregnancy. The rapid onset, wet necrotic appearance of the ulcer, dense stromal infiltration, marked anterior chamber reaction, and corneal melting were consistent with aggressive Gram-negative keratitis. The isolation of P. aeruginosa from both the corneal scrapings and contact lenses further supported the association between contact lens wear and the infection.

Pseudomonas aeruginosa causes corneal destruction through both direct microbial invasion and the production of extracellular proteolytic enzymes, particularly protease IV and P. aeruginosa small protease. These virulence factors degrade host defense proteins and stromal collagen, promote inflammatory tissue injury, and may contribute to continuing corneal melting even after the viable bacterial load has been reduced by appropriate antimicrobial treatment [3].

Treatment of infectious keratitis during pregnancy requires careful consideration of the risks associated with both maternal infection and fetal exposure to medication. Inadequately treated keratitis may result in irreversible visual loss, corneal perforation, or loss of ocular integrity. Pregnancy should therefore not result in undertreatment of a severe, sight-threatening infection.

Topically administered ophthalmic medications may enter the systemic circulation through conjunctival absorption and drainage through the nasolacrimal system into the highly vascular nasal mucosa. Although systemic exposure is substantially lower than that associated with oral or intravenous administration, it is not completely absent. Treatment decisions should therefore account for the severity of maternal disease, gestational age, expected systemic absorption, duration and intensity of treatment, and the availability of alternative agents [4].

In the present case, antimicrobial therapy was selected following consultation with the obstetric team. The immediate threat to maternal vision and ocular integrity was considered to outweigh the potential risks associated with the selected medications.

The patient’s psoriatic arthritis was in remission, and she was not receiving corticosteroids, conventional immunosuppressive agents, or biological therapy at presentation. Active systemic inflammation or treatment-related immunosuppression was therefore not considered a major contributor to the severity or persistence of the infection. Nevertheless, this history was clinically relevant because active autoimmune disease or immunomodulatory treatment could potentially influence susceptibility to infection, inflammatory responses, and corneal healing.

After 14 days of culture-directed treatment, resolution of the hypopyon and substantial reduction in anterior chamber inflammation suggested partial control of the active infection. Nevertheless, stromal infiltration and corneal melting persisted. Progressive stromal degradation may continue after a reduction in the microbial burden because of the residual activity of bacterial proteases, inflammatory cell-derived enzymes, activated keratocytes, and host matrix metalloproteinases [1,3]. Therefore, persistent melting does not necessarily indicate antimicrobial resistance or uncontrolled microbial proliferation.

Systemic doxycycline is frequently considered as an adjunctive anticollagenolytic treatment in cases of progressive corneal melting. In the present case, however, it was avoided because of the pregnancy. This limitation contributed to the decision to consider PACK-CXL as an alternative method of increasing stromal resistance to enzymatic degradation.

The decision to perform PACK-CXL was based on the persistence of structural corneal deterioration despite intensive, culture-directed antimicrobial therapy. At that stage, the treatment objective was no longer limited to reducing the microbial burden but also included preservation of corneal integrity. Emergency therapeutic penetrating keratoplasty was considered the principal rescue alternative if progressive thinning or perforation developed. Other potential rescue options included tissue adhesive application for a small perforation, amniotic membrane transplantation, and conjunctival flap surgery. However, emergency keratoplasty in an actively infected and inflamed eye during pregnancy would have introduced additional anesthetic, perioperative, postoperative, and medication-related challenges. PACK-CXL was therefore selected as a less invasive adjunctive rescue intervention before emergency transplantation became necessary.

PACK-CXL may exert both antimicrobial and biomechanical effects. Photoactivation of riboflavin by ultraviolet-A irradiation generates reactive oxygen species capable of damaging microbial nucleic acids and cellular components. At the same time, the formation of additional covalent bonds within stromal collagen and the surrounding extracellular matrix increases corneal rigidity and resistance to enzymatic digestion [3,4].

The biomechanical effect may be particularly relevant when progressive melting persists despite apparent microbiological control. By increasing stromal resistance to microbial and host-derived proteases, PACK-CXL may arrest tissue degradation and provide sufficient time for epithelial healing and resolution of the inflammatory response.

A systematic review and meta-analysis suggested that adjunctive PACK-CXL may accelerate complete corneal healing and resolution of stromal infiltration compared with standard antimicrobial treatment alone. However, the included studies demonstrated substantial clinical and methodological heterogeneity, and the certainty of the evidence was limited [8].

A Cochrane review of PACK-CXL for bacterial keratitis included only three controlled trials involving 59 eyes. The authors found very-low-certainty evidence regarding complete healing and low-certainty evidence regarding treatment failure, concluding that the effectiveness of adjunctive PACK-CXL remained uncertain [9].

In the present case, PACK-CXL was not used as a substitute for antimicrobial therapy. It was performed after 14 days of intensive, culture-directed treatment, when the hypopyon and anterior chamber inflammation had substantially improved but the stromal infiltrate and melting persisted. The subsequent arrest of stromal degradation, gradual healing of the ulcer, and avoidance of therapeutic keratoplasty suggest a potentially beneficial adjunctive effect.

Nevertheless, a direct causal relationship cannot be established from a single case. The observed improvement may have resulted from PACK-CXL, the delayed effect of antimicrobial therapy, or a combination of both interventions. The favorable outcome in this patient cannot be generalized to other patients or interpreted as establishing the efficacy of PACK-CXL.

PACK-CXL and pregnancy

It is important to distinguish elective corneal collagen cross-linking for progressive ectatic disorders from emergency or rescue PACK-CXL for sight-threatening infectious keratitis. Elective cross-linking is generally deferred during pregnancy because reproductive safety data remain limited [10]. In the present case, PACK-CXL was performed as an exceptional, off-label rescue intervention in response to persistent stromal melting and an immediate threat to corneal integrity.

Direct evidence regarding the maternal-fetal safety of CXL performed during pregnancy remains unavailable. Current United States prescribing information, revised in February 2025, states that animal developmental and reproductive studies have not been conducted with the riboflavin-ultraviolet-A cross-linking system. Because it is unknown whether the procedure can cause fetal harm or affect reproductive capacity, the prescribing information advises that CXL should not be performed during pregnancy. This recommendation reflects the absence of reproductive safety data rather than evidence of demonstrated fetal toxicity. In addition, the approved indications for this riboflavin-ultraviolet-A system are progressive keratoconus and corneal ectasia following refractive surgery. PACK-CXL for infectious keratitis, therefore, constitutes an off-label application independently of pregnancy [10].

The most recent relevant evidence remains indirect. A 2026 systematic review evaluating hormonal modulation of keratoconus included 26 studies and 4,201 participants. The review identified an association between fluctuations in hypothalamic-pituitary-gonadal axis hormones and the development or progression of keratoconus, particularly during periods of substantial hormonal change, such as pregnancy. However, it did not evaluate CXL performed during pregnancy and provided no maternal, fetal, or neonatal safety data regarding riboflavin-ultraviolet-A exposure [11]. Similarly, a prospective study of 24 eyes from 19 women who had undergone successful accelerated CXL before conception demonstrated a significant increase in maximum keratometry during pregnancy, followed by regression toward prepregnancy values after delivery [12]. These findings suggest that pregnancy-related hormonal changes may transiently influence corneal biomechanics even in previously cross-linked corneas. However, the study concerned CXL performed before conception and cannot be used to infer fetal safety when the procedure is undertaken during pregnancy.

Thus, the newer literature improves understanding of how pregnancy may influence the cornea but does not resolve the question of whether CXL can be safely performed during gestation. The absence of observed maternal or obstetric complications in the present case and the subsequent uncomplicated delivery are reassuring, but a single favorable outcome cannot establish reproductive safety or justify routine use.

PACK-CXL in a pregnant patient should consequently be considered only in exceptional, sight-threatening circumstances in which the anticipated maternal benefit outweighs the uncertain fetal risk. The decision should involve the patient, corneal specialist, and obstetric team and should consider gestational age, the severity and progression of the infection, remaining corneal thickness, response to antimicrobial therapy, risk of perforation, and the risks associated with alternative surgical treatment.

Emergency penetrating keratoplasty in an actively infected and inflamed eye would have introduced additional challenges, including anesthesia, perioperative medication exposure, recurrence of infection, graft failure, rejection, and the need for prolonged postoperative treatment. These considerations are particularly important during pregnancy, when both maternal ocular safety and potential fetal exposure must be evaluated. In the present case, stabilization of the cornea following PACK-CXL allowed preservation of ocular integrity and avoided transplantation during pregnancy.

The final visual outcome of 20/28 was favorable, particularly considering the initial visual acuity of 20/200 and the presence of progressive stromal melting. Preservation of the native cornea also avoided the long-term risks and postoperative management associated with therapeutic corneal transplantation.

## Conclusions

Adjunctive PACK-CXL was followed by complete resolution of corneal melting, healing of the ulcer, and substantial visual recovery in a pregnant patient with severe Pseudomonas aeruginosa keratitis that had responded incompletely to intensive, culture-directed antimicrobial therapy. The procedure also allowed therapeutic penetrating keratoplasty to be avoided.

PACK-CXL may represent a valuable rescue intervention in carefully selected cases of bacterial keratitis with persistent or progressive stromal melting. However, evidence regarding its effectiveness remains limited, and direct reproductive safety data are lacking. Its use during pregnancy should therefore be restricted to exceptional, sight-threatening circumstances following multidisciplinary consultation, comprehensive informed consent, and careful evaluation of the potential maternal benefits, unknown fetal risks, and risks associated with alternative surgical management.

PACK-CXL should not be considered a replacement for appropriate culture-directed antimicrobial therapy. This case illustrates a potential individualized management strategy rather than establishing a standard of care. Further studies are required to better define the safety and effectiveness of PACK-CXL in this setting.

## References

[1] Ting DS, Ho CS, Deshmukh R, Said DG, Dua HS (2021). Infectious keratitis: an update on epidemiology, causative microorganisms, risk factors, and antimicrobial resistance. Eye (Lond).

[2] Stapleton F, Carnt N (2012). Contact lens-related microbial keratitis: how have epidemiology and genetics helped us with pathogenesis and prophylaxis. Eye (Lond).

[3] Willcox MDP (2011). Pseudomonas aeruginosa infection and inflammation during contact lens wear: a review. Optom Vis Sci.

[4] Tozman EC, Gottlieb NL (1987). Adverse reactions with oral and parenteral gold preparations. Med Toxicol.

[5] Martins SA, Combs JC, Noguera G (2008). Antimicrobial efficacy of riboflavin/UVA combination (365 nm) in vitro for bacterial and fungal isolates: a potential new treatment for infectious keratitis. Invest Ophthalmol Vis Sci.

[6] McCall AS, Kraft S, Edelhauser HF (2010). Mechanisms of corneal tissue cross-linking in response to treatment with topical riboflavin and long-wavelength ultraviolet radiation (UVA). Invest Ophthalmol Vis Sci.

[7] Said DG, Elalfy MS, Gatzioufas Z (2021). Collagen cross-linking with photoactivated riboflavin (PACK-CXL) for infectious keratitis: a systematic review and meta-analysis. Cornea.

[8] Ting DS, Henein C, Said DG, Dua HS (2019). Photoactivated chromophore for infectious keratitis - corneal cross-linking (PACK-CXL): a systematic review and meta-analysis. Ocul Surf.

[9] Davis SA, Bovelle R, Han G, Kwagyan J (2020). Corneal collagen cross-linking for bacterial infectious keratitis. Cochrane Database Syst Rev.

[10] (2016). US Food and Drug Administration. Photrexa (riboflavin 5′-phosphate ophthalmic solution) prescribing information. Published.

[11] Ashraf M, Shoraka E, Shahabi S (2026). Hormonal modulation of keratoconus: a systematic review and screening strategy for at-risk populations. Ophthalmol Sci.

[12] Sarac O, Yesilirmak N, Caglayan M, Yaman D, Ozdas D, Toklu Y, Cagil N (2022). Dynamics of keratoconus progression after a previous successful accelerated crosslinking treatment during and after pregnancy. J Cataract Refract Surg.

